# Alterations in Adhesion Molecules, Pro-Inflammatory Cytokines and Cell-Derived Microparticles Contribute to Intima-Media Thickness and Symptoms in Postmenopausal Women

**DOI:** 10.1371/journal.pone.0120990

**Published:** 2015-05-19

**Authors:** Nicté Figueroa-Vega, Carmen Moreno-Frías, Juan Manuel Malacara

**Affiliations:** Department of Medical Sciences, University of Guanajuato, León campus, León, Gto., México; University of Padua, ITALY

## Abstract

Menopause, the cessation of menses, occurs with estrogens decline, low-grade inflammation, and impaired endothelial function, contributing to atherosclerotic risk. Intima-media thickness (IMT) is an early subclinical biomarker of atherosclerosis. Inflammation may have a role on symptoms: hot flashes, anxiety, and depressive mood, which also are related to endothelial dysfunction, increased IMT and cardiovascular risk. In this study we compared several inflammatory markers in early *vs*. late postmenopausal women and studied the association of IMT and symptoms with these markers in the full sample. In a cross-sectional design including 60 women (53.1±4.4 years old) at early and late postmenopause, we evaluated the expression of CD62L, ICAM-1, PSGL-1, CD11b, CD11c, and IL-8R on PBMC by flow cytometry. Serum soluble ICAM-1, sVCAM-1, sCD62E, sCD62P, CXCL8, IL-1β, IL-6, and TNF-α levels were quantified by ELISA. Plasma levels of microparticles (MPs) were determined by FACS. Finally, carotid intima-media thickness (IMT) was measured by ultrasound. We observed that ICAM-1 expression by lymphocytes and serum sVCAM-1 levels were augmented at late postmenopause. Late postmenopause women with severe hot flashes had increased expression of CD62L and IL-8R on neutrophils. By multivariate analysis, the carotid IMT was strongly associated with membrane-bound TNF-α, CD11b expression, Annexin V^+^ CD3^+^ MPs, LPS-induced NO production, HDL-cholesterol and age. Depressive mood was associated negatively with PSGL-1 and positively with LPS-induced NO. Finally, Log(AMH) levels were associated with carotid IMT, IL-8R expression and time since menopause. IMT and depressive mood were the main clinical features related to vascular inflammation. Aging, hormonal changes and obesity were also related to endothelial dysfunction. These findings provide further evidence for a link between estrogen deficiency and low-grade inflammation in endothelial impairment in mature women.

## Introduction

Menopause is the permanent cessation of menses as a result of the irreversible loss of ovarian functions with the decline of estrogen levels [1]. Women at this stage may suffer vasomotor (hot flashes and sweats), and emotional (anxiety and depressive mood) symptoms, resulting from estrogen diminution and other associated factors [2], among which low-grade chronic inflammation has been recently identified [3]. These conditions may have a severe effect in women’s quality of life, with a progressive risk for cardiovascular diseases (CVD) [4–7].

Endothelial dysfunction is a significant predictor of cardiovascular events. The age-related decline in endothelial function is delayed in premenopausal women as compared with men, but catches-up at postmenopause. Thus the perimenopausal window is a critical time for increased CVD risk [8]. A valid procedure to evaluate early cardiovascular damage is the ultrasound measurement of the carotid intima-media thickness (IMT) [9].

Endothelial dysfunction is accompanied by low-grade chronic inflammation [3, 10], loss of vascular tone and increased expression of adhesion molecules by endothelial cells (EC) such as the members of the Ig superfamily (ICAM, VCAM), integrins (CD11b), and selectins (CD62L and CD62E). These molecules facilitate the traffic of leukocytes (polymorphonuclear leukocytes, monocytes, monocytes-derived tissue macrophages, and lymphocytes) into the intima, allowing endothelial dysfunction and CVD [11, 12].

Other inflammatory biomarkers also may predict vascular events, including pro-inflammatory cytokines (IL-6, TNF-α), chemokines, and microparticles (MPs), complex vesicular structures shed by activated or apoptotic endothelial cells, leukocytes or platelet, that contain a phospholipid bilayer with transmembrane receptors [13, 14]. Reactive oxygen species (ROS) and nitric oxide (NO) [15], also participate in inflammation, destabilization of plaque, recruitment of inflammatory cells, and early atherogenesis. [16, 17].

Inflammation and vascular damage are also presumptively related to vasomotor and emotional symptoms, mainly hot flashes and depressive mood [7, 18–21], which may interact with other metabolic abnormalities frequent at menopause such as weight gain, dyslipidemia and insulin resistance [22].

Postmenopausal women may have augmented circulating markers of inflammation such as TNF-α, increasing artery sensitivity to vasoconstriction [23], as well as chemokine IL-8 levels that induce vasodilatation during hot flashes [24]. Hot flashes may be associated with IMT and inflammation [15, 25]. Yet, another report indicates that hot flashes result from impairment of endothelial function without IMT alteration [18].

Whether the impairment of endothelial function (increased IMT and altered inflammatory markers) is the consequence of the loss of endogenous estrogen or the aging process *per se*, remains unclear.

In view of this information, we compared the expression of several inflammatory markers, in groups of women at early and late postmenopause. We also analyzed the possible association of these markers with carotid artery IMT in all subjects. Additionally, considering the controversial physiopathology of symptoms at menopause we also examined the relationship of inflammation at menopause, with hot flashes, emotional symptoms and hormone levels.

## Materials and Methods

### Study population

Sixty healthy postmenopausal women were recruited by home visits and invitations in public places in León, Mexico. Menopausal status was established by the cessation of menses for at least 1 year in regularly menstruating women. Women were categorized according to Stages of Reproductive Aging Workshop (STRAW) criteria [26] as early (≤5 yr) or late (>5 yr) postmenopause. Early menopause indicates mainly the influence of estrogen deprivation. On the other hand, late menopause shows the influence of aging with the impairment of vascular function, dyslipidemia and tissue deterioration.

For inclusion we considered non-hysterectomized, non-pregnant, non-lactating, normotensive (resting blood pressure <140/90 mm Hg) women, who did not have clinical evidence of neoplastic, chronic infectious, cardiovascular diseases, or antecedents of arrhythmias nor hormone alterations. They did not receive anxiolytic, antidepressants, hypnotic medication, β-blockers, Ca channel blockers, anti-hypertensives, statins, and fibrates or hormone replacement therapy in the previous 6 months.

In this study we compared early postmenopausal with late postmenopausal women in order to examine differences on changes attributable to recent estrogen deprivation with changes derived from chronic organic deterioration.

A written informed consent was obtained from all volunteers. This study was approved by the Institutional Bioethics Committee from Department of Medical Science, University of Guanajuato, León Campus.

### Anthropometric data

We collected the age of women, schooling in years, exercise at least 1 day/week (yes or no), smoking habits (yes or no), and alcohol consumption for at least once per week (yes or no), date of the last menstrual period, age at menarche, menstrual history, and numbers of deliveries. Height and weight were obtained with indoor clothing, without shoes, and body mass index (BMI) (kg/m^2^) was calculated. Waist circumference was measured at the midpoint between the lowest rib and the iliac crest. Blood pressure was obtained with a random zero sphygmomanometer in sitting position after 5 min rest.

### Evaluation of menopausal symptoms

Women answered a symptoms’ questionnaire validated and used in previous works by our group [2, 27] to assess hot flashes, depressive mood, and anxiety.

*Hot flashes* were registered according to *intensity* as none = 0, slight = 1 (<5 min duration, resolved without disturbing daily life), moderate = 2 (<15 min, disturbing only at daytime), severe = 3 (>15 min, disrupting daily life and sleep).

*Depressive mood* was evaluated using the Hamilton-Bech-Rafaelsen Scale [28] including nine items: sadness, problems with work and occupation, fatigue, non-systematized pain, sense of guilt, diminished verbal activity, suicidal tendencies, slow thinking, and altered sleep. The items are rated from 0 to 4 in terms of frequency categories (never, sometimes, often and always). Total score (range: 0 to 26) was obtained by adding the values for each response.

The *anxiety* score was obtained using an 18-item self-report questionnaire regarding breathlessness, palpitation, tremor, agitation and fear to madness, with each item scored as yes (1) or no (0). Total scores ranged from 0 to 18.

### Carotid artery intima-media thickness (IMT) measurement

Carotid artery IMT measurements were carried out using an Echo-Doppler ultrasound (Acuson X150; Siemens Medical Solutions, Munich, Germany) with a 7 MHz VF10-5 linear transducer, including pulsed and color Doppler. The color Doppler was used to identify the carotid artery. Measurements were made in a region 1–2 cm proximal to the carotid bifurcation, and the intima-media thickness (IMT) of the far wall was evaluated as the distance between the lumen-intima interface and the media-adventitia interface. For analysis the average for right and left carotid artery was calculated. The digitized M-mode ultrasonographic records were analyzed offline using a computer program in which the images were recalled and magnified. For each image, measurements were performed during one cardiac cycle, which was divided into 10 equal sections.

The presence of plaque was determined for each of the 5 segments of the left and right carotid artery (distal and proximal CCA, carotid bulb, and proximal internal and external carotid artery). Plaque was defined as a distinct area protruding into the vessel lumen at least 50% thicker than the adjacent IMT. This analysis categorized plaque as either absent or present. Two observers reviewed all measurements.

### Blood samples

Blood samples were drawn from the antecubital vein in a sitting position after overnight fasting for 12 hours. Serum was separated and stored at -80°C until use. Salivary cortisol was evaluated with two samples obtained 600 to 800 hours and the second one 1800 to 2000 hours. Saliva samples were centrifuged to obtain the supernatant, which was a low viscosity specimen. All samples were taken in accordance with the regulations and approval of the Institutional Review Board of the Department of Medical Sciences, University of Guanajuato, León Campus.

### Clinical and laboratory evaluation

For serum lipids determination, we used standard methods. Glucose concentrations were analyzed by an enzymatic method (glucose dehydrogenase; Olympus Diagnostica GmbH, Hamburg Germany).

Serum 17β-estradiol (E_2_) (ESTR-CTRIA RIA kit, Cisbio Bioassays, Fr), FSH levels (ImmunoChem FSH-CT IRMA, ICN Biomedicals, Inc, CA), as well as, salivary cortisol concentrations (CORT-CT2 RIA Kit, Cisbio Bioassays, Fr), were measured by RIA, following the manufacturer’s instructions. The normal ranges are shown in Table 1. Anti-Müllerian hormone (AMH) was determined by ELISA (MyBiosource) with a detection limit of 0.375 ng/ml. Intra- and interassay coefficients of variation were 3.5%, and 4.9% for 17β-E_2_, and 5.4% and 4.6% for FSH. All analyses were carried out by duplicate.

**Table 1:** Comparison of anthropometric data, hormones and symptoms between early and late postmenopausal women.

	Early postmenopausal women mean±SD	Late postmenopausal women mean±SD	t	*p*-value
**Age (yr)** ^**a**^	52.4±4.2	54.9±4.7	-2.0	0.052
**Years since menopause (yr)**	3.0±1.4	10.9±4.3	-10.8	<0.0001^***^
**Age at menarche (yr)**	12.6±1.3	13.3±2.0	-1.4	0.18
**Number of Parity**	3.8±2.3	5.3±2.9	-2.0	0.053
**BMI (kg/m** ^**2**^ **)** ^**a**^	28.4±4.5	30.3±8.0	-1.2	0.24
**Glucose (mg/dl)** ^**a**^	94±10	95±9	-0.6	0.52
**Total cholesterol (mg/dl)** ^**a**^	191±29	208±35	-1.9	0.06
**HDL-cholesterol (mg/dl)** ^**a**^	60±6	60±5	0.1	0.94
**Triglycerides (mg/dl)** ^**a**^	158±62	206±83	-2.4	<0.019^*^
**FSH (mIU/ml**)^**a**^	86.2±40.2	84.1±49.5	0.2	0.87
**17β-E2 (pg/ml)**	6.5±10.9	5.0±4.9	0.72	0.47
**AMH (ng/ml)**	3.3±4.3	3.5±1.5	-0.1	0.88
**Salivary diurnal cortisol (nmol/L)**	1.9±1.1	1.0±0.0	0.8	0.45
**Salivary evening cortisol (nmol/L)**	3.0 ±1.9	2.2±0.0	0.4	0.68
**IMT (mm)**	0.4±0.1	0.4±0.1	0.6	0.54
**Depressive mood (score 0–26)** ^**a**^	6.1±3.8	7.1±5.4	-0.8	0.42
**Anxiety (score 0–18)** ^**a**^	8.7±5.2	9.3±5.6	-0.4	0.70
**Hot flashes intensity (score 0–3)** ^***b***^	**N(%)**	**N(%)**	**χ** ^**2**^	***p***
**0 (Nule)**	10(36.6)	7(23.3)	3.5	0.06
**1 (Slight)**	2(6.6)	11(38.8)	0.06	0.08
**2 (moderate)**	12(40)	4(13.3)	5.8	<0.016^*^
**3 (severe)**	12(40)	2(6.6)	3.1	0.08

All variables are shown as mean ± SD or percentage, as appropriate. Group means were compared by Student’s t test.

^*a*^ Statistics calculated on logarithmic transformed values.

^*b*^ Proportions were analyzed using chi-squared (*X* ^2^) test.

^*^ *p*<0.05

^**^ *p*<0.01

^***^ *p*<0.001.

Reference ranges at menopause: 17β-E2, <35pg/ml; FSH, 40–200 mIU/ml; morning cortisol, 6.2–38.1 nmol/L; evening cortisol, 0.6–4.9 nmol/L.

BMI, body mass index; HDL, high-density lipoprotein; AMH anti-Müllerian hormone; FSH, follicle-stimulating hormone; IMT; intima-media thickness.

### Measurement of inflammation markers in serum

Serum concentrations of soluble P-selectin, sE-selectin, sVCAM, sICAM-1, CXCL8/IL-8, IL-1β, IL-6, and TNF-α were measured using ELISA kits (R&D Systems, Minneapolis, USA) according to the manufacturer’s instructions. The lower detection limits for sP-selectin, sE-selectin, sVCAM-1, sICAM-1, CXCL8/IL-8, IL-1β, IL-6, and TNF-α were 0.5, 0.009, 0.6, and 0.96 ng/ml, 3.5, 1.0, 0.039, and 0.106 pg/ml, respectively. All analyses were carried out by duplicate.

### Cell isolation

Peripheral blood mononuclear cells (PBMC) were isolated from heparinized blood by density-gradient centrifugation over Ficoll Hypaque (density 1.077 g/ml; Sigma-Aldrich, St. Louis, MO). Next, neutrophils were isolated by sedimentation over Dextran 1.3% (Sigma). Cell viability was determined by trypan blue dye exclusion, and it was always greater to 95%.

### Flow cytometry analysis

Cell surface markers expression on neutrophils was evaluated by simple immunofluorescence staining with the following monoclonal antibodies (mAbs):-CD62L-FITC (L-selectin),-IL-8R-FITC (CD182/CXCR2), all purchased from BD (San José, CA) and-CD11b (Bear) kindly provided by Dr. Sánchez-Madrid (Madrid, Spain). In the case of PBMC we employed following mAbs:-CD62L-FITC,-CD54-PE (ICAM-1), and-PSGL-1-PE (CD162), In addition, we explored the expression of integrins on monocytes with next mAbs:-CD11c-PECy5 (all of them from BD), and-Bear-1 (CD11b) (Madrid, Spain). As negative staining controls, isotype matched irrelevant mAbs were used (BD). Cell analysis was performed with a FACSCanto II (Becton Dickinson, USA) flow cytometer. Data were subsequently analyzed using the FACSDiva software (BD Biosciences, San Diego, CA) and results were expressed as the percent of positive cells and/or the mean fluorescence intensity (MFI).

### Measurement of ROS

Neutrophils were incubated with 2´7´-dichlorofluorescein diacetate (Sigma, St. Louis, MO) during 15 minutes at 37°C, in presence of PMA (10 μg/ml). Finally, ROS production was analyzed in a FACSCanto II flow cytometer (Becton Dickinson). The results are expressed as the delta of MFI.

### Isolation of microparticles (MPs)

A venous blood sample was collected in a heparinized syringe. Immediately after collection, cells were removed by centrifugation to obtain platelet free plasma. This plasma was then transferred to a new tube and the supernatant was discarded. Pellet MPs were resuspended in 500 μl of Annexin V binding buffer (eBiosciences, San Diego, CA). For flow cytometric analysis, 80 μl of MPs suspension was used per tube. The MPs suspension were stained with Annexin V-FITC (eBiosciences) and labeled with anti-CD3,-CD14,-CD41 or-CD62E-PE-conjugated mAbs (from BD Biosciences). Finally, MPs were resuspended in 300 μl and analyzed in a FACSCanto II flow cytometer (Becton Dickinson, San Jose, CA, USA), using FACSDiva software (Becton Dickinson). To set the gate of the MPs we used polystyrene latex beads (0.2 μm and 1.0 μm, Sigma-Aldrich) and as negative control used unlabeled MPs. The MPs were defined as double positive stained for Annexin V-FITC and cell surface marker-PE. The number of MP/μl of plasma was calculated according to Berckmans et al [29] with the following formula: MP/l = n x (100 μl/5 μl) x (950 μl/V) x (106/250 μl), where n = absolute number of MP determined by FACS analysis; 100 μl = total volume of washed microparticles suspension; 5 μl = pellet used for analysis; 950 μl = total volume before analysis (pellet + antibodies + buffer); 250 μl = original volume of the sample before isolation of MP.

### Assay of nitrites

PBMC were cultivated at 2 x 10^5^ cells/ml in absence and presence of lipopolysaccharide (LPS) (1000 ng/ml; Sigma), during 48 hours. Supernatants were harvested and frozen until determination of nitrites/nitrates or NO metabolites (NOx) using Griess method. For each assay a calibration curve was made with a stock of sodium nitrate 250μM.

### Statistical analysis

Data are shown as the mean±SD or percentage. Normality was assessed with the Kolmogorov-Smirnov test. We compared groups of women at early- *vs* late postmenopause, using T tests for independent samples for normally distributed variables. For non-parametric data, differences were analyzed after logarithmic transformation. Kruskal-Wallis test and *post-hoc* comparisons were carried out using the Dunn´s test for the analysis according to menopause stage and hot flashes intensity. Categorical variables were analyzed using the *χ* ^2^ test

Multiple stepwise regression analysis was carried out in the whole group of study to identify factors associated with IMT, symptoms and hormones. For symptoms we tested as candidate regressors: inflammation markers and hormones. For hormone levels we tested: inflammatory markers and symptoms; finally, for IMT we tested symptoms, inflammatory molecules and hormones as regressors. In this manner, we constructed a model via the stepwise inclusion of significant factors (Model #1). After a significant model was obtained, these analyses were repeated including as confounding factors BMI, waist girth, total cholesterol, HDL-cholesterol, age, and years since menopause (Model #2). Exercise, smoking habit, and alcohol consumption were not tested as possible confounding factors, because few women smoke (n = 6), none of them consumed alcohol regularly, or made exercise. In all analyses, we applied a normal distribution model.

Analyses were carried out using the Statistica 5.0 (Stat Soft Inc, Tulsa, OK), and GraphPad Prism 6.0 (San Diego, CA) softwares. *p*<0.05 was considered statistically significant.

## Results

### Characteristics of postmenopausal women

Volunteers had a mean age of 53 years and 5.1 years since menopause, mainly with excess weight, but with normal glucose and cholesterol levels. Only six women reported smoking habit.

Moderate or severe hot flashes were reported by 50% of women. The scores for depressive mood and anxiety were moderately elevated in each group.

As expected, in full sample levels of FSH were high (85.6±42.5 mIU/ml) and estradiol low (6.8±11.7 pg/ml). The serum concentrations of AMH were also low.

Mean IMT values for the group of postmenopausal women were within the normal range (<0.4 mm, but a S.D. of 0.1 indicated that about 18% of the subjects had more than 0.5 mm). Finally, we did not observe any women with atheroma plaque formation.

### Comparison between early and late postmenopausal women

Table 1 shows the comparison of women at early and late postmenopause. Women at early menopause (n = 36) were marginally younger (54.4±4.2 years) than late postmenopausal women (n = 24) (54.9±4.7 years). Means of years since menopause were 3.0 and 10.9 years respectively for early and late postmenopause. This difference may permit, at late postmenopause show the influence of other factors related to tissue deterioration, such as chronic inflammation, dyslipidemia, and obesity.

Women at late postmenopause had increased BMI values displaying an obesity profile, and lower values of E_2_, however IMT was not significantly different. Although, we observed diminished morning salivary and evening cortisol concentrations in late postmenopausal women, the difference was not significant (p>0.05).

### ICAM-1 expression is augmented on lymphocytes but CD62L^+^ lymphocytes were decreased at late postmenopause

**Fig 1:**
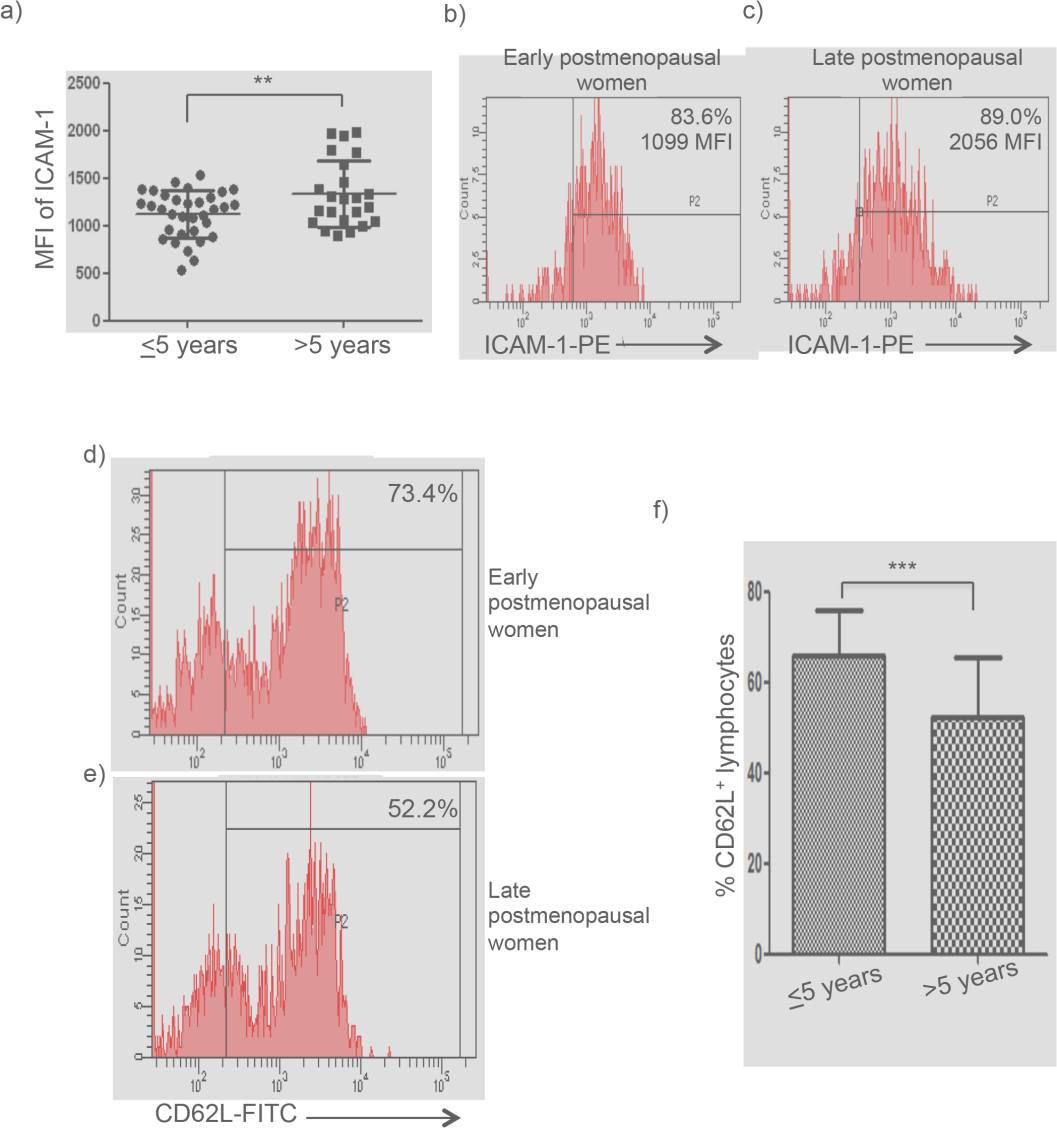
Overexpression of ICAM-1 by lymphocytes and decreased of CD62L^+^ cells from late postmenopausal women. Mononuclear cells were isolated from the peripheral blood and were analyzed by flow cytometry for the adhesion molecules expression in lymphocytes gate. **a)** Single data points of ICAM-1 expression by lymphocytes are shown from both groups of postmenopausal women. ICAM-1 expression was significantly higher in late postmenopause women. Mean group values were compared by Student´s T test. **b-c)** Flow cytometry analysis of ICAM-1 expression on lymphocytes from early and late postmenopausal women. Histograms correspond to ICAM-1 staining from an early postmenopausal woman and a late postmenopausal woman. Percentages of positive cells and MFI values are indicated. **d-e)** Representative flow cytometry analysis percent of CD62L^+^ peripheral lymphocytes from an early and a late postmenopausal women are shown. Percentages of marked-positive cells are indicated. CD62L^+^ cells were significantly higher in early postmenopausal women. **f)** Data of percent of CD62L^+^ peripheral lymphocytes from early and late postmenopausal women are shown as mean±SD. (**p<0.01; ***p<0.001). MFI (mean fluorescence intensity).

We found a slight significant increase of ICAM-1 expression on freshly isolated lymphocytes from the group at late postmenopause compared to that at early postmenopause (p = 0.01) (Fig 1A–1C and Table 2).

**Table 2:** Comparison of inflammatory biomarkers between early and late postmenopausal women.

	Early postmenopausal women mean±SD	Late postmenopausal women mean±SD	t	*p*-value
***Microparticles***				
*Annexin V* ^*+*^ *MPs (MPs/L)* ^a^	2875±3674	2653±1423	0.2	0.83
*Annexin V* ^*+*^ *CD41* ^*+*^ *MPs (MPs/L)*	952±1440	643±442	0.8	0.45
*Annexin V* ^*+*^ *CD3* ^*+*^ *MPs (MPs/L)* ^a^	2057±3799	1851±1929	0.2	0.85
*Annexin V* ^*+*^ *CD62E* ^*+*^ *MPs (MPs/L)* ^a^	2285±4029	1821±1670	0.4	0.69
*Annexin V* ^*+*^ *CD14* ^*+*^ *MPs (MPs/L)* ^a^	4609±3529	4384±3801	0.2	0.84
***Inflammatory markers on monocytes***				
*% CD14* ^*+*^ *CD11b* ^*+*^ *monocytes*	80±14	75±12	1.3	0.2
*% CD14* ^*+*^ *CD11c* ^*+*^ *monocytes*	98±4	95±6	2.5	<0.016^*^
*CD11b (MFI)*	970±657	1146±751	-0.9	0.38
*CD11c (MFI)* ^*a*^	2988±1783	2226±990	1.6	0.11
*Membrane-bound TNF-α MFI)*	548±356	1060±1341	-2.3	<0.027^*^
*Reactive oxygen species (ROS) (delta)*	2.0±1.0	2.0±1.2	0.2	0.87
*Nitric oxide production (μM)*	337±512	532±579	-1.3	0.21
*LPS-induced nitric oxide production (μM)*	313±556	652±871	-1.7	0.08
***Inflammatory markers on lymphocytes***				
*% ICAM-1* ^*+*^ *lymphocytes* ^*a*^	71±12	68±16	0.9	0.35
*% CD62L* ^*+*^ *lymphocytes* ^*a*^	66±10	52±13	4.2	<0.0001^***^
*% PSGL-1* ^*+*^ *lymphocytes* ^*a*^	94±4	94±6	0.2	0.88
*ICAM-1 (MFI)* ^*a*^	1235±411	1375±310	-1.2	<0.05^*^
*CD62L (MFI)* ^*a*^	2737±919	2366±1025	1.3	0.18
*PSGL-1 (MFI)* ^*a*^	14936±6143	17384±6858	-1.3	0.2
***Inflammatory molecules on neutrophils***				
*% CXCR2 (IL-8R)* ^*+*^ *neutrophils*	91.5±13.2	89.0±19.3	0.6	0.57
*% CD62L* ^*+*^ *neutrophils*	93.4±7.5	93.4±6.8	-0.0	1.0
*% CD11b* ^*+*^ *neutrophils*	84.4±18.0	76.9±23.5	1.3	0.2
*CD62L (IMF)*	1886±1055	1604±645	1.0	0.32
*CXCR2 (IL-8R) (MFI)*	1605±1311	1081±534	1.5	0.13
*CD11b (MFI)*	1216±1693	1431±2131	-0.4	0.69
***Soluble molecules***				
*sICAM-1 (ng/ml)* ^*a*^	282.6±105.8	290.1±76.3	-0.3	0.79
*sVCAM-1 (ng/ml)* ^*a*^	710.8±279.4	867.8±286.4	-1.9	0.05^*^
*sE-Selectin (ng/ml)* ^*a*^	56.2±19.3	46.6±24.4	1.6	0.11
*sP-selectin (ng/ml)* ^*a*^	64.5±49.3	74.7±58.6	-0.7	0.5
*CXCL8 (IL-8) (pg/ml)*	3.5±0.0	6.6±12.5	-1.7	0.10
*IL-1β (pg/ml)*	28.8±19.4	26.5±21.0	0.4	0.69
*IL-6 (pg/ml)*	2.2±2.1	2.2±2.0	-0.1	0.89
*sTNF-α (pg/ml)*	6.7±6.9	6.2±5.2	0.3	0.79

All variables are shown as mean ± SD. Means between groups were analyzed by T student test.

^*a*^ Statistics calculated on logarithmic transformed values.

*p*<0.05 was considered significant.

^*^ *p*<0.05

^**^ *p*<0.01

^***^ *p*<0.001

Annexin V^+^MPs, MP positive for phosphatidyl serine; Annexin V^+^CD3^+^MPs, lymphocytes-derived MPs; Annexin V^+^CD14^+^MPs, monocytes-derived MPs; Annexin V^+^CD62E^+^MPs, endothelial-derived MPs; Annexin V^+^CD41^+^MPs, platelet-derived MPs; sICAM-1, soluble intercellular adhesion molecule-1; sVCAM, soluble vascular cell adhesion molecule-1; sP-Selectin, soluble platelet-selectin, sE-Selectin; soluble endothelial selectin; sL-Selectin, soluble lymphocyte selectin; PSGL-1, P- and E-Selectin glycoprotein ligand-1; TNF-*α*, tumoral necrosis factor-alfa; IL-8R, interleukin-8 receptor; ROS, reactive oxygen species; NO, nitric oxide; LPS, lipopolysaccharide; IMT; intima-media thickness; MFI, mean fluorescence intensity.

In addition, the percent of CD62L^+^ peripheral lymphocytes were diminished in late postmenopausal women (p<0.001) (Fig 1D–1F and Table 2).

No significant differences were found for other adhesion molecules between both groups (p>0.05).

### TNF-α was overexpressed on monocytes at late postmenopause, but the percentage of CD14^+^CD11c^+^ cells was diminished at late postmenopause

**Fig 2:**
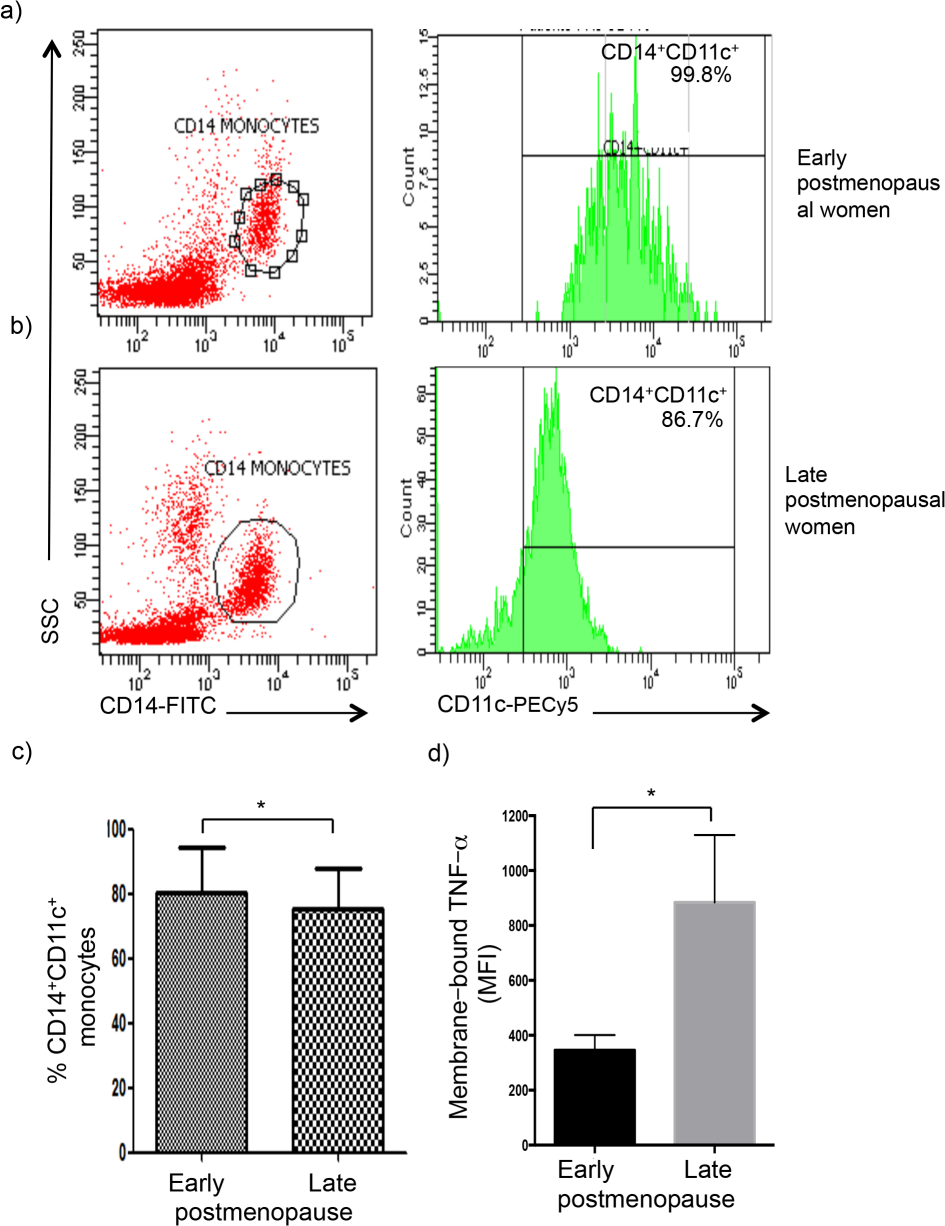
Alterations in membrane-bound TNF-α-expression and percent of CD14^+^CD11c^+^ monocytes at late postmenopause. Mononuclear cells were isolated from the peripheral blood and immunostained for CD11c and membrane-bound TNF-*α* in CD14^+^monocytes gate, and analyzed by flow cytometry. **a-b)** Green histograms correspond to CD14^+^CD11c^+^ monocytes. Percentages of marker-positive cells are indicated. Data shown are representative of an early and a late postmenopausal woman. **c)** Percent of CD14^+^CD11c^+^ monocytes in early postmenopause was significantly higher than late postmenopause. Data shown as the mean±SD. *p<0.05, Student´s T test. **d)** Comparison of membrane-bound TNF-*α* expression by monocytes from early and late postmenopausal women is shown as the mean±SD. Mean group values were compared by Student´s T test. (*p<0.05).

We also observed that the percent of CD14^+^CD11c^+^ monocytes was lower at late postmenopause (p<0.02) (Fig 2A–2C and Table 2). However, late postmenopausal women had significantly elevated expression of membrane-bound TNF-α on peripheral CD14^+^ monocytes (p<0.03) (Fig 2D and Table 2). The expression of the remaining molecules was not significantly different between groups (p>0.05).

### Soluble adhesion molecules were slightly increased at late postmenopause

**Fig 3:**
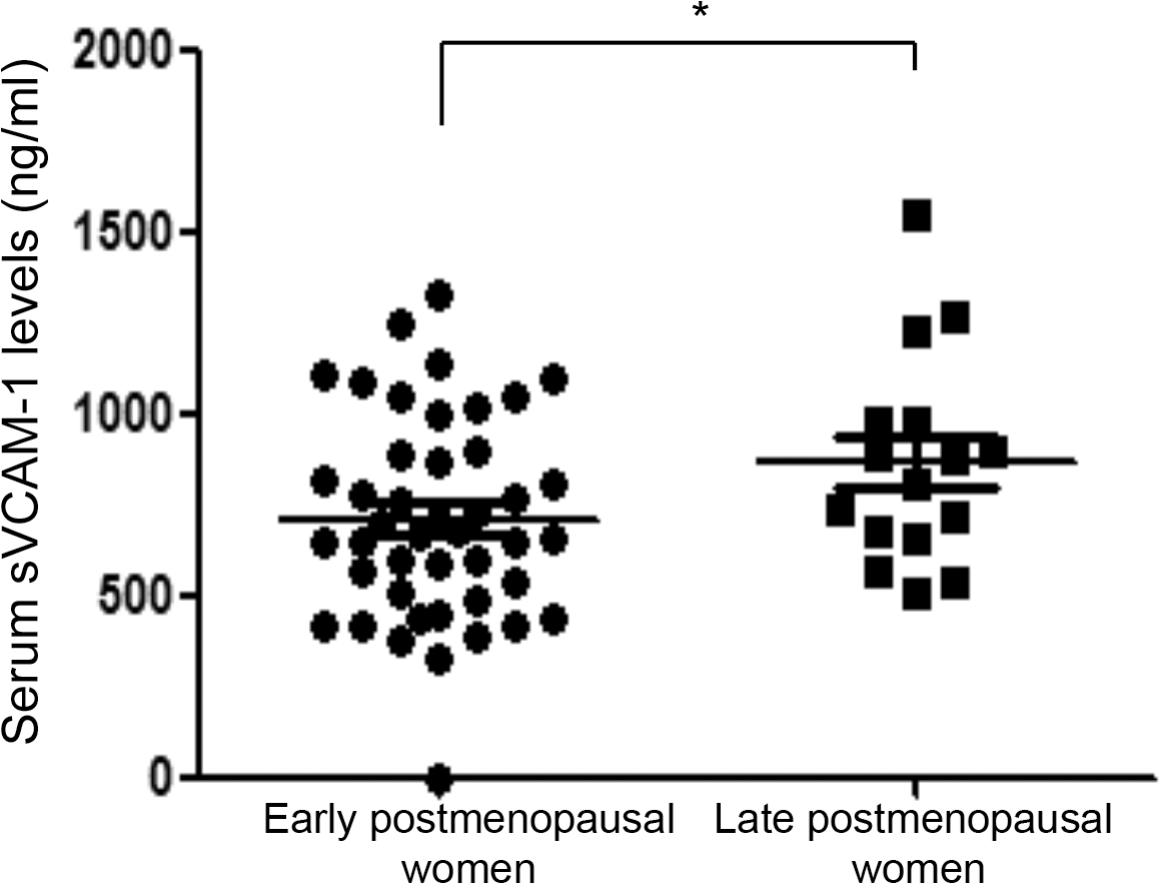
Serum levels of sVCAM-1 are increased in late postmenopause. sVCAM-1 levels were determined by ELISA in serum samples from early and late postmenopausal women. Data are shown as the median and interquartile range. (*p<0.05).

The concentrations of sVCAM-1 were increased in late postmenopausal women in comparison those with early postmenopause (p = 0.05) (Fig 3 and Table 2), but remaining soluble molecules were not different in both groups (p>0.05).

### Adhesion molecules were overexpressed by neutrophils in late postmenopausal women with hot flashes

**Fig 4:**
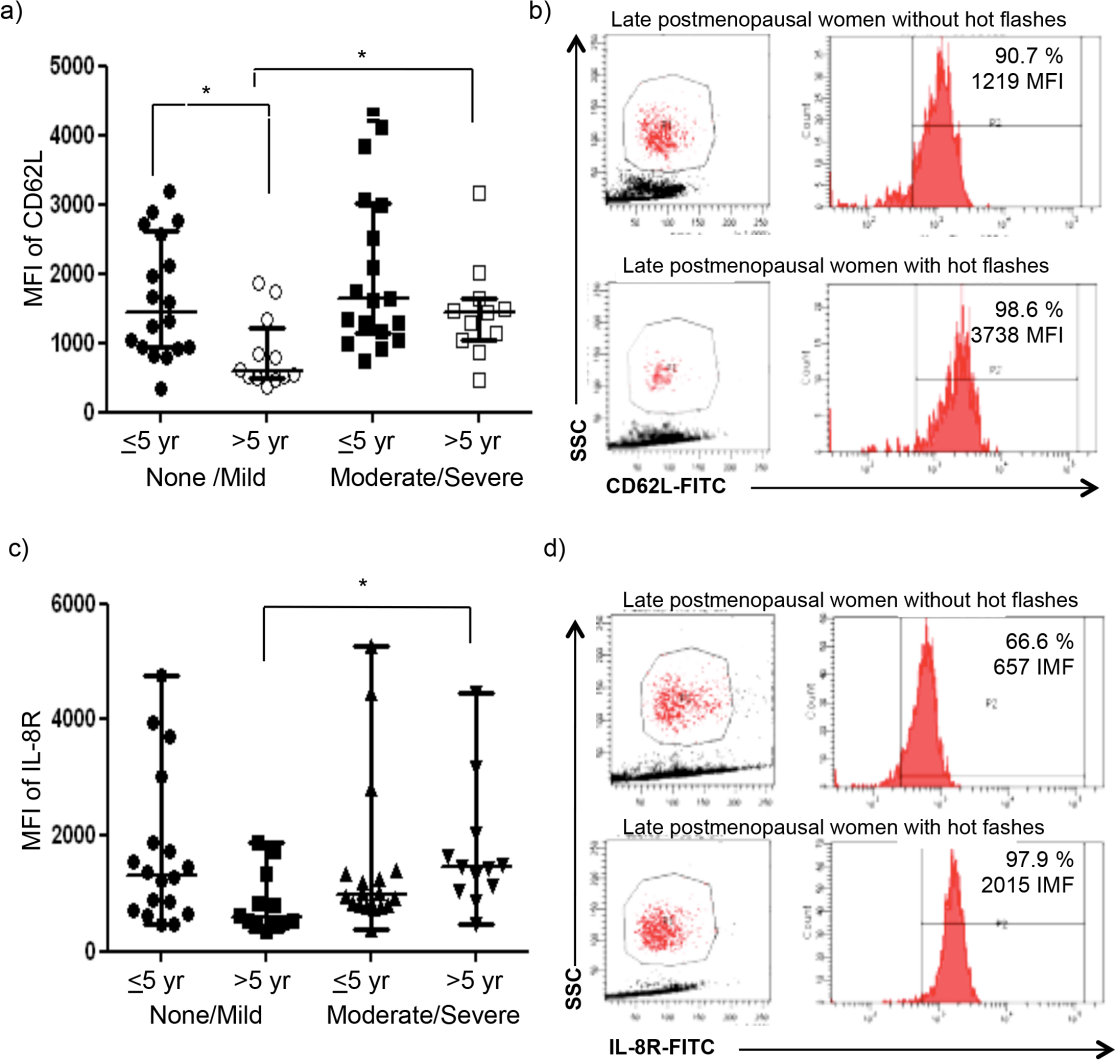
CD62L and IL-8R expression is increased on neutrophils from postmenopausal women with hot flashes. **a-b)** Neutrophils were isolated as stated in Material and Methods, then labeled for CD62L or **c-d)** IL-8R, and the percent of cells and MFI values were determined by flow cytometry analysis. Data are shown as the median and interquartile range. *p<0.05, Kruskal-Wallis test and *post hoc* comparisons were carried out by Dunn´s test. Representative dot plots and histograms are shown. Red histograms correspond to CD62L **(b)** or IL-8R **(d)** staining. Percentages of positive cells and MFI values are indicated. MFI (mean fluorescence intensity).

In further analysis, we divided both groups (early and late postmenopause) by severity of hot flashes, and then we compared the differences between groups. As shown in Fig 4, CD62L expression by neutrophils was higher in early postmenopausal women without hot flashes as compared with late postmenopausal women without hot flashes (p<0.05) (Fig 4A). At late postmenopause, CD62L expression by neutrophils was increased in women with moderate and severe hot flashes (p<0.05) (Fig 4A and 4B).

Finally, late postmenopausal women with moderate or severe hot flashes displayed an increased IL-8R expression by neutrophils in comparison with women without hot flashes (p = 0.05) (Fig 4C and 4D).

### Several inflammation markers were associated with carotid IMT in postmenopausal women

The results of variables associated with carotid artery IMT, symptoms (depressive mood, anxiety and hot flashes), and hormones by multiple regression analysis are shown in Table 3.

**Table 3:** Association of inflammatory markers with symptoms, carotid artery IMT, and hormones according to a multiple regression procedure.

Dependent variable	Regressor	Beta ± S.E.	T	*p*-value
**Carotid artery IMT**				
Model #1 Adjusted R² = 0.532				
	***Membrane-bound TNF-α***	0.32 ± 0.10	3.35	<0.001^***^
	***Annexin V*** ^***+***^ ***CD3*** ^***+***^ ***MPs***	0.36 ± 0.10	3.60	<0.001^***^
	***CD11b expression***	0.86 ± 0.17	4.98	<0.001^***^
	***LPS-induced NO production***	0.58 ±0.14	4.11	<0.001^***^
	***ICAM-1 expression***	-0.38 ± 0.12	-3.16	<0.003^**^
	***CD62L expression***	0.34 ± 0.14	2.50	<0.015^*^
Model #2 Adjusted R² = 0.531				
	***Membrane-bound TNF-α***	0.33 ± 0.09	3.54	<0.001^***^
	***Annexin V*** ^***+***^ ***CD3*** ^***+***^ ***MPs***	0.28 ± 0.10	2.82	<0.007^**^
	***CD11b***	0.52 ± 0.11	4.81	<0.001^***^
	***LPS-induced NO production***	0.28 ± 0.11	2.58	<0.012^*^
	***Age***	-0.22 ± 0.09	-2.37	<0.021^*^
**Depressive mood**				
Model #1 Adjusted R² = 0.193				
	***PSGL-1 expression***	-0.03±0.01	7.29	<0.007^**^
	***LPS-induced NO production***	0.000±0.000	8.39	<0.004^**^
**Hot flashes intensity**				
Model #2 Adjusted R² = 0.100				
	***BMI***	-0.34±0.12	-2.75	<0.008^**^
**Log (AMH)**				
Model #1 Adjusted R² = 0.124				
	***Carotid artery IMT***	0.27 ±0.122	2.23	<0.03^*^
	***sE-Selectin levels***	-0.26±0.122	-2.14	<0.03^*^
Model #2 Adjusted R² = 0.193				
	***Carotid artery IMT***	0.29 ± 0.12	2.47	<0.016^*^
	***Years since menopause***	0.32 ± 0.12	2.72	<0.009^**^
	***IL-8R (CD182/CXCR2)***	0.23±0.12	1.99	0.050^*^
**Log (FSH)**				
Model #1 Adjusted R² = 0.199				
	***LPS-induced NO production***	-0.37 ± 0.12	-3.09	<0.003^**^
	***Hot flashes intensity***	0.29 ± 0.12	2.50	<0.015^*^
	***CD62L expression***	-0.28 ± 0.12	-2.31	<0.024^*^
Model #2 Adjusted R² = 0.239				
	***BMI***	-0.40 ± 0.12	-3.45	<0.001^***^
	***Annexin V*** ^***+***^ ***MPs***	-0.27 ± 0.12	-2.28	<0.026^*^
	***LPS-induced NO***	-0.25±0.12	-2.21	<0.031^*^
**Log (17β-Estradiol)**				
Model #1 Adjusted R² = 0.086				
	***IL-6 concentrations***	0.31±0.12	2.56	<0.013^*^
Model #2 Adjusted R² = 0.267				
	***Age***	-0.36±0.11	-3.14	<0.003^**^
	***BMI***	0.30±0.12	2.63	<0.011^*^

Multiple regression in all whole group (n = 60).

*p*<0.05 was considered statistical significant.

^*^ *p*<0.05

^**^ *p*<0.01

^***^ *p*<0.001

Model #1 without and Model #2 with testing for confounding variables: group.

BMI, body mass index; AMH anti-Müllerian hormone; FSH, follicle-stimulating hormone; PSGL-1, P- and E-Selectin glycoprotein ligand-1; sTNF-α, soluble tumoral necrosis factor-alfa; NO, nitric oxide; LPS, lipopolysaccharide; IMT; intima media-thickness; Annexin V^+^CD3^+^ MPs, lymphocytes-derived MPs; Annexin V^+^ MPs, MP positive for phosphatidyl serine; S.E., standard error.

The intima-media thickness (IMT) showed a strong, and positive associations with membrane-bound TNF-α, Annexin V^+^ CD3^+^ MPs plasma levels, CD11b expression by monocytes, LPS-induced NO production (p<0.01 in all cases), and CD62L expression by lymphocytes (p<0.02); but association was negative with ICAM-1 expression (p<0.01). However, no hormone or symptom was included in the analysis. Model #2, testing confounding factors, included age (p<0.03) and HDL-cholesterol (p>0.05), with a marginally inverse association.

### Inflammatory biomarkers had a relationship with depressive mood, but neither with hot flashes nor anxiety

As shown Table 3, the intensity of hot flashes was not associated with any inflammatory marker, and after testing for confounding factors, a marginal negative association was found with BMI (p<0.05).

Depressive mood was negatively associated with PSGL-1 expression by lymphocytes (p<0.01), and positively with LPS-induced NO concentrations (p<0.02). These associations were not altered after testing for possible confounding factors.

Anxiety was not associated with any hormones or inflammation markers.

### Various inflammation markers and IMT correlated with hormones in postmenopausal women

The study of factors associated with hormone levels using multiple regression analysis showed log(AMH) associated positively with carotid IMT (p<0.03), but negatively with sE-Selectin (sCD62E (p<0.05). In model #2, remained the association with carotid IMT (p<0.02), and years since menopause was now included (p<0.01), appearing a marginal association with IL-8R (p>0.05) (Table 3).

We found log(FSH) negatively associated with LPS-induced NO production (p<0.003), and with CD62L expression by lymphocytes (p<0.03), but positively with hot flashes (p<0.02). Model #2, adjusted for confounding factors, showed a strong negative association with BMI, with Annexin V^+^ microparticles, and LPS-induced NO (Table 3).

Finally, log(17β-E_2_) was associated positively with IL-6 serum levels (p<0.02). However, Model #2, showed associations positive with BMI (p<0.006), and a negative with age (p<0.003) (Table 3).

## Discussion

Endothelial dysfunction is an early alteration of CVD risk, which in women is evident only after menopause, suggesting that estrogen may play a key role in maintaining a healthy and functional endothelium. In this regard, inflammatory infiltrates may cause thickening of the involved arterial vessel wall leading to progressive stenosis, atherosclerosis and occlusion. Activated macrophages and T lymphocytes are fundamental elements in vascular inflammation.

To our knowledge, this study is the first attempt to test a comprehensive list of inflammatory markers related to endothelial dysfunction, considering leukocyte-endothelial cell interactions and circulating levels of cytokines and chemokines, and explore their associations with carotid artery IMT, symptoms and hormones at early and late postmenopause.

The univariate comparison of women at early and late menopause revealed that women at late postmenopause exhibit a decrease of CD14^+^CD11c^+^ cells and CD62L^+^ lymphocytes, but an enhanced membrane-bound TNF-α expression on CD14^+^ monocytes, as well as increased adhesion molecules expression. The decrease of CD14^+^CD11c^+^ monocytes and CD62L^+^ lymphocytes is explained by the fact that aging individuals have decreased immune function. This condition is named immune-senescence that may result from suppressive factors secreted by macrophages [30]. In contrast, the augmented membrane-bound TNF-α expression in CD14^+^ monocytes, could be associated with the progression of estrogen deprivation, which further impairs the immune function, facilitating inflammation, increasing leukocyte adhesiveness to the endothelium and plaque formation [11].

In regards to adhesion molecules expression, several reports show that ICAM-1 expression is increased at postmenopause [31, 32], as found in our study. Likewise, the adhesion molecule sVCAM-1 is also overexpressed by activated endothelium, which promotes the recruitment and extravasation of leukocytes. ICAM-1 and VCAM-1 expression is up regulated during endothelial-dysfunction at postmenopause, a phenomenon also influenced by estrogen receptors (ERs) SNPs [33], contributing to systemic inflammation and endothelial damage. Thus the impaired endothelial function is more evident after prolonged estrogen deficiency during the postmenopausal years, as shown in our results.

In the univariate comparison of hormones and symptoms at early and late postmenopause we found similar FSH levels. This confirms that the elevation of FSH occurs since early postmenopause, and the decrease at advanced age is moderate. In addition, gonadotropin levels at menopause are very pulsatile [34], therefore its quantification is highly variable in ranges of minutes. The hormonal conditions for estradiol and AMH were also similar at early and late post-menopause. The scores for symptoms were also similar in both groups.

Aging is a dynamic and complicated process with an important effect in vascular functions in which participates estrogen decrease. With aging occur changes in the immune system, destabilizing vascular endothelium and up-regulation of inflammatory molecules, permitting deterioration and atherogenesis. Our findings suggest that the endothelium is also important in the decline in immune function, given its central role in the inflammatory response. Women at early postmenopause, have less than 5 years since the last menses, which may results because of their younger age or a later age at menopause. This means a shorter time with estrogen deprivation. In contrast women with five or more years since menopause, are at greater risk of endothelial dysfunction and inflammation. For this process the discrimination of the effect of older age and more years without significant estrogen exposure may not be reliable.

Various studies demonstrate that IMT rise continuously with age, yet, others establish that IMT increase at late perimenopause and progressively diminish at postmenopause [35, 36], in agreement with our data. Therefore, that explains that IMT may not progress with the advancement of menopause. As show our findings, IMT had a normal mean value (0.4 mm) but with a S.D. of 0.1, about 18% of the subjects should have increased levels. Thus, evaluation of IMT in multivariate analyses is useful for determining factors involved in cardiovascular risk.

The multivariate study of factors related to IMT, showed a strong positive association with Annexin V^+^ CD3^+^ MPs levels, CD11b expression, LPS-induced NO production, membrane-bound TNF-α, HDL-cholesterol levels and age. It is of interest the strong association of carotid IMT with lymphocyte-derived microparticles (Annexin V^+^CD3^+^ MPs). Previous reports found platelet-microparticles associated with IMT and other atherosclerosis risk factors in obese subjects and menopausal women [13, 14]. These elements contain markers of lymphocyte cells induced for activation or apoptosis. MPs are diffusible vectors of specific molecules and cytokines promoting cellular interactions and signal transmission. Our findings suggest that they may be used for clinical diagnosis of vascular damage at postmenopause, because lymphocytes-derived microparticles may have paracrine functions, influence atherosclerosis and are also markers of lipid-rich atherosclerotic plaques.

The relationship between IMT and the augmented expression of TNF-α in monocytes agrees with the pivotal role of TNF-α in inflammation [11], since macrophages secrete and express TNF-α as a reaction to danger. This initiates the immune response, including increased leukocyte adhesiveness to the endothelium, thus facilitating its recruitment to the inflammation sites. This process is a major component of the atherosclerotic plaque formation. Classically activated (M1) macrophages contribute to plaque instability. In addition, increased cholesterol in arteries facilitates the infiltration of monocytes into plaque formation.

NO is an atheroprotector molecule, therefore, altered NO production may increase atherosclerotic lesions. The diminished dilatation is the first manifestation of endothelial dysfunction. We found a positive correlation between IMT and the *in vitro* synthesis of NO by monocytes. We consider that this finding deserves further investigation; yet, it is feasible to speculate that the increased ability to synthesize a vasodilator molecule (NO) might be a compensatory phenomenon in patients with chronic endothelial dysfunction.

In our study IMT also was negatively associated with HDL-cholesterol levels, an important factor for reverse cholesterol transport. This supports the concept that IMT progression is favored by impaired removal of cholesterol from macrophage foam cells, for transportation to the liver [37].

Another inflammatory factor associated with carotid IMT was CD11b, an α-integrin, involved in attachment and migration of leukocytes during endothelial activation contributing to atherosclerosis and cardiovascular risk [15]. Abu-Taha, et al [3] reported that CD11b expression is up regulated in circulating monocytes from postmenopausal women. Gomita et al, also found increased expression of CD11b at perimenopause [38], suggesting that this molecule is important at this stage and could be a useful biomarker.

It is of note the strong association of inflammation markers with carotid IMT, but not with hot flashes or emotional symptoms in our study. This does not support a direct association of hot flashes with risk for vascular damage, as reported in previous works [22, 36], but agree with the report of Ohira et al, [7] who also showed IMT associated with the anger score but not with anxiety or depression. Therefore, our work suggests that carotid artery IMT changes may be independent of symptoms, and it is possible that IMT may be related with other metabolic and emotional factors.

At late postmenopause, women with moderate to severe hot flashes had an activated phenotype of neutrophils that expressed IL-8R (CXCR2). The role of neutrophils on atherothrombotic disease is well established. Interestingly, postmenopausal women have increased IL-8 serum levels associated with peripheral vasodilatation [24], which may participate in hot flashes. As shown in the multivariate regression model #2, the main regressor for hot flashes was BMI. This finding may be explained by increased stress response frequently found in obese subjects [39]. Although, we did not find any inflammatory marker associated with hot flashes, as reported by others [40], currently the physiopathology of hot flashes favors two mechanisms: the participation of inflammation, and the hypothalamic autonomic imbalance [41].

Depressive mood showed an unexpected strong negative association with PSGL-1, which may be involved in endothelial damage. PSGL-1 (P-Selectin glycoprotein ligand-1, or CD162), is expressed in activated T lymphocytes, and plays an important role in tethering and rolling of activated endothelial cells and atherosclerosis plaque formation [42]. A recent work shows increased PSGL-1-expressing T lymphocytes in perimenopausal women, and it is down regulated by estrogen therapy [38]. We are not aware of other reports on PSLG-1 in depressive mood at postmenopause; therefore this subject requires further studies.

The relationship of depressive mood with NOx suggests that probably excess of NO metabolites may act as toxic molecules. Women at menopause frequently suffer depression, with altered Hypothalamic-Pituitary-Adrenal axis [21]. We interpreted these changes to be part of the neurohumoral response to depression. In contrast to previous studies, we did not find anxiety correlated with inflammation molecules [43, 44].

In regards to factors associated with hormones, the duration of menopause was strongly associated with AMH levels, which is considered as an indicator of ovarian follicular reserve [45]. It is possible that AMH is also under the influence of metabolic and inflammatory factors. The strong association of AMH with carotid artery thickness may result of indirect relationships of extraovarian roles of AMH.

We found interleukin-8 receptor (IL-8R or CD182) with a weak and positive association to AMH. IL-8 is a chemokine involved in ovulation and may be influenced by sex steroid hormones [46]. This suggests that AMH influences the IL-8-IL-8R interactions at menopause.

The association of FSH with LPS-induced NO may be explained by a direct effect of chronic NO deficiency on FSH and LH release [47]. FSH association with hot flashes results from estrogen deprivation [48]. When BMI was tested as a confounding variable, a strong negative association with FSH was shown, revealing the estrogenization that usually results from obesity. Our data on inverse relationships between FSH levels with CD62L and Annexin V^+^ MPs agrees with the immune function decline after estrogen deprivation [30].

Finally, 17βE_2_ deficiency induces mild increases in pro-inflammatory cytokines, such as IL-1, IL-6, and TNF-α, enhancing an inflammatory process during menopause [49]. On the other hand, the correlation with phosphatidyl-serine positive microparticles may be explained by the increase of circulating microparticles in asymptomatic menopausal women with declining 17*β*-estradiol levels [50]. These cellular changes may explain in part the progression of cardiovascular disease. Therefore, the assessment of circulating microparticles expressing phosphatidyl-serine may be used for the detection of inflammatory environment at menopause.

**Fig 5:**
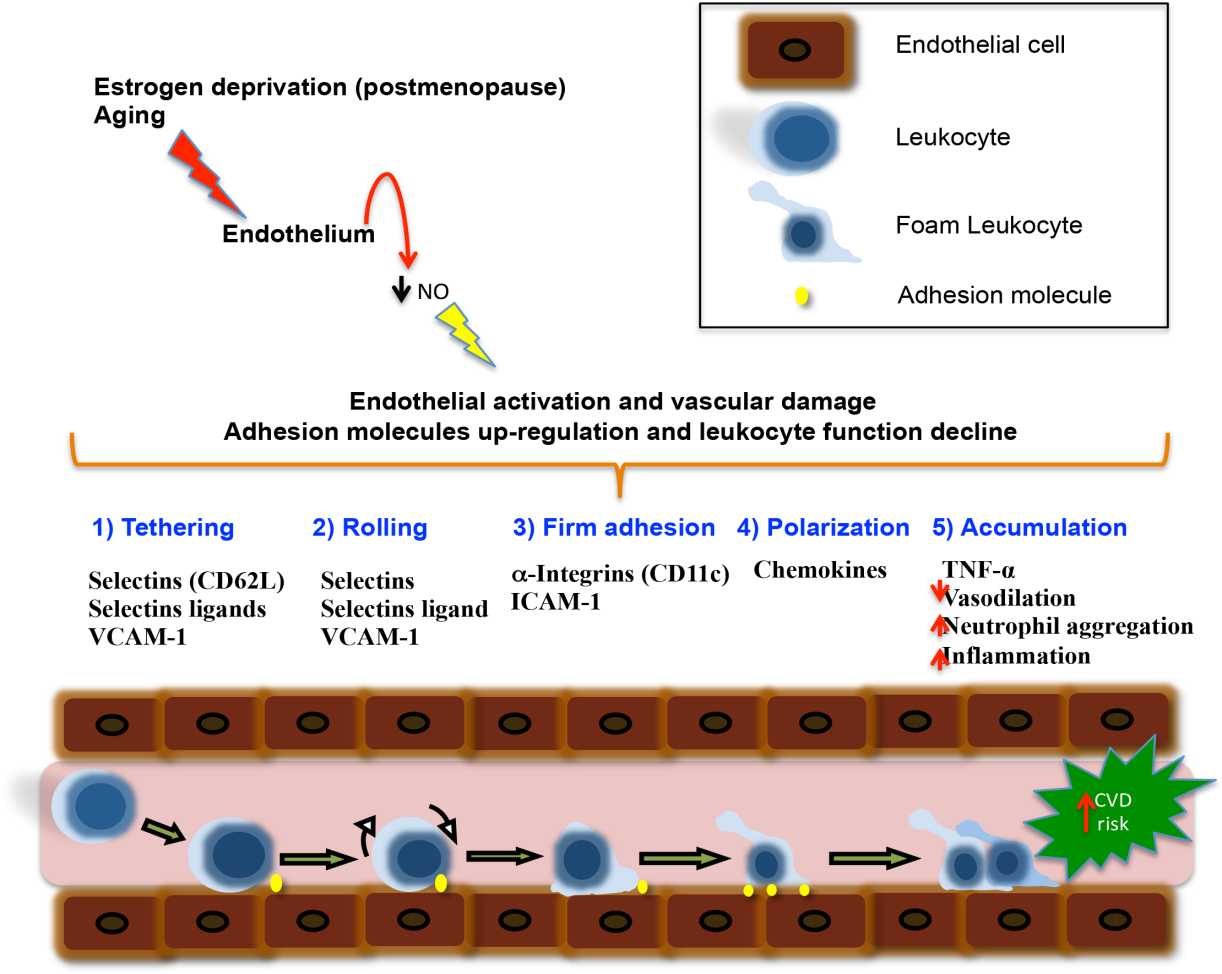
Possible effects of estrogens deprivation and aging on inflammation and endothelial damage at menopause. When estrogens decline and during old age, important changes occur in the endothelium: first, estrogen decrease, leads a low NO bioavailability inducing destabilization of endothelial cells, favoring up-regulation of adhesion molecules. Under such conditions, aged vessels have a compromised vasodilation, inducing endothelial dysfunction, increased vascular resistance and impaired tissue perfusion. Moreover, the atherosclerosis process is accompanied by a low-grade inflammation, which could be favored by endothelial dysfunction. The recruitment of leukocytes into areas of inflammation is mediated by interacting sets of cell adhesion molecules (showed step by step in order of activation). T-cells and monocytes are the first cells infiltrating the arterial intima, and represent a hallmark of endothelial damage favoring the early stages of atherogenesis. In atherosclerosis, focal expression of key adhesion molecules, mainly triggered by plasma atherogenic lipoproteins, may also mediate the recruitment of mononuclear cells to the plaque. Among these adhesion molecules, ICAM-1, a protein of the Ig superfamily, and one of the ligands for LFA-1, has been suggested to play an important role in atherogenesis. Thus, the increased ICAM-1 expression detected in this study could be associated with an enhanced recruitment of monocytes and T lymphocytes. Finally, in advanced stages, immune cells function also decline, contributing to inflammation and vascular damage and triggering CVD risk. (Modified from González-Amaro R. [51]). ICAM-1, soluble intercellular adhesion molecule-1; sVCAM, soluble vascular cell adhesion molecule-1; L-Selectin, soluble lymphocyte selectin; TNF-α, tumoral necrosis factor-alfa; IL-8R, interleukin-8 receptor; NO, nitric oxide.

We are aware of the limitations of cross-sectional and observational studies, in which a causal association cannot be supported. In addition the sample size does not permit identify others associations. However, the strong positive associations for carotid IMT with inflammation markers supports further studies on the role of pro-inflammatory molecules and its possible use as early diagnostic tool of endothelium damage at menopause, and for the detection of an inflammatory environment in depressive mood at postmenopause. The role of pro-inflammatory molecules in diverse biological problems is currently in evolution; and one of the most enigmatic fields is its role on CVD risk, and especially the interaction of hormone conditions at menopause. As shown in Fig 5 [51], we emphasize the role of estrogen deprivation (menopause) and aging, in development of chronic inflammation and endothelial function impairment at midlife. Positive and negative findings must be confirmed with new studies, and interpreted in the future considering new physiopathological findings.

Additional information of the endothelial damage may be obtained other measures such as pulse wave velocity (aPWV), and braquial artery flow-mediated dilation (FMD). Those tests, provide further evidence, but are no substitute the IMT evaluation.

## Conclusions

In summary, our findings have important clinical implications, since we have detected that pivotal vascular inflammation molecules, involved in the early and late events of endothelial dysfunction, were elevated at postmenopause stage, contributing thus to atherosclerosis risk. In addition, our data also show carotid IMT strongly associated with markers of inflammation and endothelial dysfunction, but not with hot flashes. FSH, 17β-estradiol and AMH levels also were correlated with vascular inflammation.

## Supporting Information

S1 Dataset Complete data from early and late postmenopausal women. S1 Dataset shows the individual data for every women in the study, including anthropometric data, gynecological history, symptoms, hormones, IMT measurement, and vascular damage and inflammation markers.
(XLS)
